# Artificial intelligence for the detection of COVID-19 pneumonia on chest CT using multinational datasets

**DOI:** 10.1038/s41467-020-17971-2

**Published:** 2020-08-14

**Authors:** Stephanie A. Harmon, Thomas H. Sanford, Sheng Xu, Evrim B. Turkbey, Holger Roth, Ziyue Xu, Dong Yang, Andriy Myronenko, Victoria Anderson, Amel Amalou, Maxime Blain, Michael Kassin, Dilara Long, Nicole Varble, Stephanie M. Walker, Ulas Bagci, Anna Maria Ierardi, Elvira Stellato, Guido Giovanni Plensich, Giuseppe Franceschelli, Cristiano Girlando, Giovanni Irmici, Dominic Labella, Dima Hammoud, Ashkan Malayeri, Elizabeth Jones, Ronald M. Summers, Peter L. Choyke, Daguang Xu, Mona Flores, Kaku Tamura, Hirofumi Obinata, Hitoshi Mori, Francesca Patella, Maurizio Cariati, Gianpaolo Carrafiello, Peng An, Bradford J. Wood, Baris Turkbey

**Affiliations:** 1 Molecular Imaging Branch, National Cancer Institute, National Institutes of Health, Bethesda, MD USA; 2 Clinical Research Directorate, Frederick National Laboratory for Cancer Research, Frederick, MD USA; 3 State University of New York-Upstate Medical Center, Syracuse, NY USA; 4 Center for Interventional Oncology, Radiology and Imaging Sciences, NIH Clinical Center and National Cancer Institute, Center for Cancer Research, National Institutes of Health, Bethesda, MD USA; 5 Radiology and Imaging Sciences, Clinical Center, National Institutes of Health, Bethesda, MD USA; 6 NVIDIA Corporation, Bethesda, MD USA; 7 Philips Research North America, Cambridge, MA USA; 8 Center for Research in Computer Vision, University of Central Florida, Orlando, FL USA; 9 Department of Radiology Fondazione IRCCS Cà Granda, Ospedale Maggiore Policlinico Milano, Milan, Italy; 10 Diagnostic and Interventional Radiology Service, ASST Santi Paolo e Carlo, San Paolo Hospital, Milan, Italy; 11 Postgraduation School in Radiodiagnostics, Università Degli Studi di Milano, Via Festa del Perdono 7, 20122 Milan, Italy; 12 Self-Defense Forces Central Hospital, Tokyo, Japan; 13 Department of Health Sciences, University of Milano, Milan, Italy; 14 Department of Radiology, Xiangyang NO.1 People’s Hospital Affiliated to Hubei University of Medicine Xiangyang, Hubei, China

**Keywords:** Infectious diseases, Computer science

## Abstract

Chest CT is emerging as a valuable diagnostic tool for clinical management of COVID-19 associated lung disease. Artificial intelligence (AI) has the potential to aid in rapid evaluation of CT scans for differentiation of COVID-19 findings from other clinical entities. Here we show that a series of deep learning algorithms, trained in a diverse multinational cohort of 1280 patients to localize parietal pleura/lung parenchyma followed by classification of COVID-19 pneumonia, can achieve up to 90.8% accuracy, with 84% sensitivity and 93% specificity, as evaluated in an independent test set (not included in training and validation) of 1337 patients. Normal controls included chest CTs from oncology, emergency, and pneumonia-related indications. The false positive rate in 140 patients with laboratory confirmed other (non COVID-19) pneumonias was 10%. AI-based algorithms can readily identify CT scans with COVID-19 associated pneumonia, as well as distinguish non-COVID related pneumonias with high specificity in diverse patient populations.

## Abstract (web summary)

Chest CT is emerging as a valuable diagnostic tool for clinical management of COVID-19 associated lung disease. Here, the authors present a multinational study on the application of deep learning algorithms for COVID-19 diagnosis against multiple lung conditions as controls.

## Introduction

Coronavirus Disease 2019 (COVID-19) has become a global pandemic with an exponential growth rate and an incompletely understood transmission process. The virus is harbored most commonly with little or no symptoms, but can also lead to a rapidly progressive and often fatal pneumonia in 2–8% of those infected^1–3^. The exact mortality, prevalence, and transmission dynamics remain somewhat ill-defined in part due to the unique challenges presented by SARS-CoV-2 infection, such as peak infectiousness at or just preceding symptom onset and a poorly understood multi-organ pathophysiology with dominant features and lethality in the lungs^4^. The rapid rate of spread has strained healthcare systems worldwide due to shortages in key protective equipment and qualified providers^5^, partially driven by variable access to point-of-care testing methodologies, including reverse transcription polymerase chain reaction (RT-PCR). As rapid RT-PCR testing becomes more available, challenges remain, including high false negative rates, delays in processing, variabilities in test techniques, and sensitivity sometimes reported as low as 60–70%^6,7^.

Computed tomography (CT) is a test that provides a window into pathophysiology that could shed light on several stages of disease detection and evolution^7–9^. While challenges continue with rapid diagnosis of COVID-19, frontline radiologists report a pattern of infection that is somewhat characteristic with typical features including ground glass opacities in the lung periphery, rounded opacities, enlarged intra-infiltrate vessels, and later more consolidations that are a sign of progressing critical illness. While CT and RT-PCR are most often concordant^9^, CT can also detect early COVID-19 in patients with a negative RT-PCR test^9^, in patients without symptoms, or before symptoms develop or after symptoms resolve^10,11^. CT evaluation has been an integral part of the initial evaluation of patients with suspected or confirmed COVID-19 in multiple centers in Wuhan China and northern Italy^12–15^. A recent international expert consensus report supports the use of chest CT for COVID-19 patients with worsening respiratory status or in resource constrained environments for medical triage of patients who present with moderate–severe clinical features and a high pretest probability of COVID-19^16^. However, these guidelines also recommend against using chest CT in screening or diagnostic settings in part due to similar radiographic presentation with other influenza-associated pneumonias. Techniques for distinguishing between these entities may strengthen support toward use of CT in diagnostic settings.

Due to the rapid increase in number of new and suspected COVID-19 cases, there may be a role for artificial intelligence (AI) approaches for the detection or characterization of COVID-19 on imaging. CT provides a clear and expeditious window into this process, and deep learning of large multinational CT data could provide automated and reproducible biomarkers for classification and quantification of COVID-19 disease. Prior single center studies have demonstrated the feasibility of AI for the detection of COVID-19 infection, or even differentiation from community acquired pneumonia^17,18^. AI models are often severely limited in utility due to homogeneity of data sources, which in turn limits applicability to other populations, demographics, or geographies. This study aims to develop and evaluate an AI algorithm for the detection of COVID-19 on chest CT using data from a globally diverse, multi-institution dataset. Here we show robust models can be achieve up to 90% accuracy in independent test populations, maintaining high specificity in non-COVID-19 related pneumonias, and demonstrating sufficient generalizability to unseen patient populations/centers.

## Results

### Patient cohorts for training and testing

**Fig. 1:**
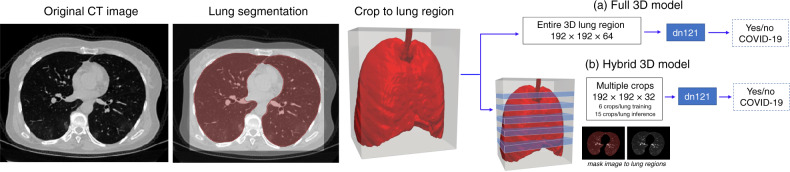
3D classification workflow. All CT images under lung segmentation for localization to chest cavity region. Following cropping to lung region, two methods were considered for differentiation of COVID-19 from other clinical entities. **a** Full 3D Model resampled the cropped lung region of CT to a fixed size (192 × 192 × 64 voxels) for input to algorithm. **b** Hybrid CT resampled the cropped lung region of CT to fixed resolution (1mm × 1mm × 5mm) and sampled multiple 3D regions (192 × 192 × 32) for input to algorithm. At training, 6 regions/patient were used. At inference 15 regions/patient were used and results were averaged to produce final probability of COVID-19.

**Fig. 2:**
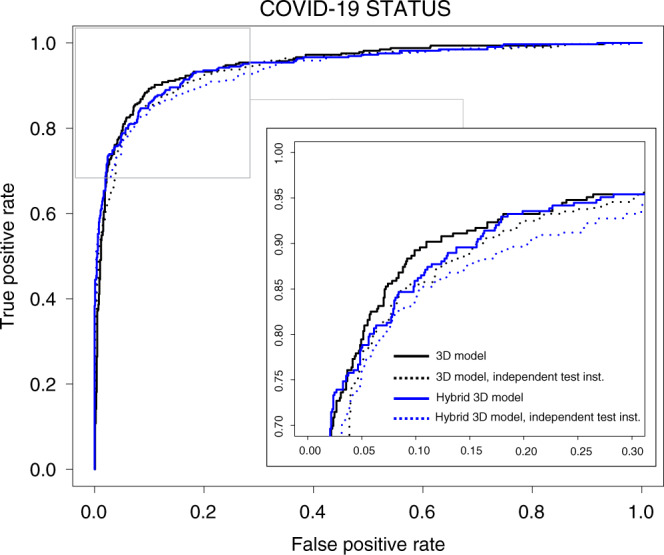
Model performance. Receiver operating characteristic (ROC) curve for 3D and hybrid 3D classification models. Both experimental conditions are shown, with highlighted area to zoom in at upper left area of the curve. Solid lines represent original training design, dotted lines indicate independent testing population design.

In total, 2724 scans from 2617 patients were used in this study, including 1029 scans of 922 patients with RT-CPR confirmed COVID-19 and lung lesions related to COVID-19 pneumonia. This includes one scan from one patient who was confirmed to have COVID-19 from the SUNY cohort. Of these, 1387 scans from 1280 patients were utilized for algorithm development, and 1337 patients were utilized for algorithm testing and evaluation. The split of data in training, validation, and test datasets can be seen in Table 1. Prevalence of COVID-19 patients in the testing set was 24.4% (326/1337). During training, all CTs for a given patient under conditions described above were included. For testing evaluation, one scan series per patient was considered. In conditions where patients underwent multiple CT scans, the initial positive CT with RT-PCR confirmed disease were used. Two classification models were developed for further evaluation (Fig. 1), one utilizing the entire lung region with fixed input size (full 3D) and one utilizing average score of multiple regions within each lung at fixed image resolution (hybrid 3D). Training converged at highest validation accuracy of 92.4% and 91.7% for hybrid 3D and full 3D classification models, respectively, for the task determining COVID-19 vs. other conditions. Overall performance is shown in Table 2. The highest test set accuracy was observed with the 3D classification model (90.8%), with resultant probability of COVID-19 disease demonstrating 0.949 AUC (Fig. 2).

**Table 1:** Patient cohorts utilized in model development and testing. Demographic values are reported as absolute numbers for patient sex and as median (range) for patient age.

Disease cohort	Center	Demographics	Training	Validation	Testing
COVID-19	Hubei, China	363 Male, 353 female Median 49 (18^a^–92)	369 Scans *354 Patients*	122 Scans *113 Patients*	207 Scans *207 Patients*
	Milan, Italy	220 Male, 90 female Median 60 (18–96)	57 Scans *52 Patients*	24 Scans *17 Patients*	54 Scans *54 Patients*
	Tokyo, Japan	91 Male, 60 female Median 60 (4–87)	100 Scans *45 Patients*	31 Scans *15 Patients*	49 Scans *49 Patients*
	Milan, Italy	10 Male, 5 female Median 55 (31–85)	–	–	15 Scans *15 Patients*
	Syracuse, NY, USA	^b^See footnote	–	–	1 Scan *1 Patient*
Any clinical indication	Syracuse, NY, USA	437 Male, 534 female Median 65 (19–100)	356 Scans *356 Patients*	93 Scans *93 Patients*	500 Scans *500 Patients*
Cancer diagnosis and/or staging	LIDC^23^	N/A	149 Scans *149 Patients*	50 Scans *50 Patients*	271 Scans *271 Patients*
	NIH, USA	100 Male Median 69 (30–89)	–	–	100 Scans *100 Patients*
Pneumonia	Syracuse, NY, USA	73 Male, 42 female Median 66 (13–101)	–	–	140 Scans *140 Patients*
	NIH, USA	28 Male, 8 female Median 21 (4–71)	28 Scans *28 Patients*	8 Scans *8 Patients*	–
Total			*1059 Scans* *984 Patients*	*328 Scans* *296 Patients*	*1337 Scans* *1337 Patients*

^a^Age was not readily available for all Hubei, China patients.

^b^Demographics for COVID-19 diagnosis from SUNY is included in all-comer/any clinical indication grouping.

**Table 2:** Performance of 3D and hybrid 3D classification models for two experimental conditions.

Design	Model	Validation accuracy	Test summary stats					
			ACC	SENS	SPEC	PPV	NPV	AUC
Original training schema	3D	0.917	0.908	0.840	0.930	0.794	0.948	0.949
	Hybrid 3D	0.924	0.889	0.853	0.901	0.735	0.950	0.947
Independent testing population	3D	0.939	0.896	0.845	0.916	0.793	0.939	0.941
	Hybrid 3D	0.905	0.895	0.751	0.951	0.853	0.909	0.938

Original training design included 1337 patients in testing cohort (of which, *n* = 326 patients with COVID-19 positivity). Independent testing population design included 1397 patients in testing cohort (entire patient cohort from Tokyo, Japan excluded from training/validation), with a total of *n* = 386 patients with COVID-19 positivity.

*ACC* accuracy, *SENS* sensitivity, *SPEC* specificity, *PPV* positive predictive value, *NPV* negative predictive value, *AUC* area under the curve.

### Algorithm performance evaluation by disease entity

Model classification accuracy was evaluated individually by disease cohort (Supplementary Table 1). All models demonstrated mixed performance in the second Italian Hospital (accuracy 53–60%), which was excluded from training and validation in both experimental conditions. Of 15 patients in this dataset, 10 patients demonstrated high disease burden and advanced, bilateral consolidating pneumonia by expert radiologist evaluation. Misclassification rates in control patients was lowest in patients undergoing CT for oncologic staging and workup (ranging 3.8–5.5% in SUNY, LIDC, NIH datasets) compared with patients with laboratory confirmed pneumonias (10%) and general population of patients undergoing CT as part of clinical care, ranging 2.7–27.3% for general evaluation to acute/trauma-related care (Supplementary Table 2). False positive findings in the cohort of patients with pneumonia further varied by etiology, with 13.7% in bacterial (7/51), 16.7% in fungal (3/18), and 4.9% in viral (3/61) infections.

**Fig. 3:**
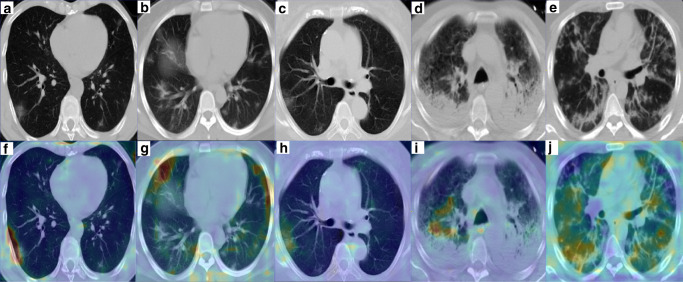
Grad-CAM* resultant saliency maps for five representative COVID-19 patients from testing set. All images are of correctly predicted positive by 3D model. Within the heatmap, areas of red indicate activation of the algorithm related with COVID-19 prediction. **a**, **b** Images and (**f**, **g**) associated maps from Hubei, China cohort. **c** Image and (**h**) associated map from Tokyo, Japan cohort. **d** Image and (**i**) associated map from an advanced case in Milan, Italy Center #1. Note activation in non-consolidating areas for prediction of COVID-19, indicating specific features independent of pneumonia-related consolidation are learned. **e** Image and (**j**) associated map of an advanced case in Milan, Italy Center #2. Note: case (**e**) represents an unseen testing center from training/validation centers. *footnote: Grad-CAM images are produced from preprocessed input data, including cropping to lung region and resizing to fixed dimension, which may result in visible changes to anatomic aspect ratio.

In COVID-19 positive cases from the test set, specificity of AI-based findings with COVID-19 lung disease were assessed by visual evaluation and review of Grad-CAM mappings. Visualization of region-based activation features from the 3D model are shown for representative test set patients in Fig. 3. Review of these Grad-CAM based maps demonstrate consistent activation in peripheral regions of the lung with COVID-19 associated disease across variable amounts of disease burden.

### Algorithm performance in unseen population domain conditions

To assess the utility of these models for COVID-19 sensitivity at independent institutions, the cohort of COVID-19 patients from Tokyo, Japan were removed from training and validation datasets and models were retrained utilizing identical algorithm configuration and hyperparameters as the original models. Overall, validation and testing accuracy were stable between models trained with and without patients from the leave-out institution (Table 2), with a modest decrease in AUC (Fig. 2). However, sensitivity in the hybrid 3D model decreased to 75%. Specifically, within patients from the independent testing center, the 3D classification model correctly identified 87/109 patients as having COVID-19 associated CT findings while the hybrid 3D classification model correctly identified 74/109 patients. Evaluation of accuracy, specificity, and sensitivity as a function of AI-based output from the 3D model demonstrated consistent model behavior at all likelihood of COVID-19 related cut points (Supplementary Fig. 1), showing model performance decreased consistently by 5–10% compared with the entire testing cohort of COVID-19 positive patients. In hybrid 3D model, lowering the cutoff probably from 0.5 to 0.376 increased the sensitivity of the model performance to that of the 3D model, though specificity decreased from 95.1 to 92.8% (Supplementary Fig. 2).

## Discussion

Preliminary studies indicate chest CT has a high sensitivity for detection of COVID-19 lung pathology and several groups have demonstrated the potential for AI-based diagnosis, reporting as high as 95% detection accuracies^17–20^. Implementation of these AI efforts at new institutions are hampered by the tendency for AI to overfit to training populations, including technical bias from institutional-specific scanners to clinical population bias due to regional variation in the use and timing of CT. Therefore, this study was specifically designed to maximize the potential for generalizability. The hypothesis was that an algorithm trained from a highly diverse multinational dataset will maintain sufficient performance accuracy when applied to new centers, compared with algorithms trained and testing in only one center. To achieve this, COVID-19 CT scans were obtained from four hospitals across China, Italy, and Japan, where there was a wide variety in clinical timing and practice for CT acquisition. Such CT indications included screening-based settings (i.e., fever clinics), where patients underwent CT the same day as initial positive PCR (China), but also included advanced disease, such as inpatient hospitalization settings at physician’s discretion (Italy). Furthermore, the inclusion of patients undergoing routine clinical CT scans for a variety of indications including acute care, trauma, oncology, and various inpatient settings was designed to expose the algorithm to diverse clinical presentations. Here we achieved 0.949 AUC in a testing population of 1337 patients resulting in 90.8% accuracy for classification of COVID-19 on chest CT.

The use of CT scans for the purpose of diagnosing COVID-19 pneumonia has been somewhat controversial^16^. In Hubei Province, China, CT scans were used extensively and at presentation, in an effort to quickly diagnose, isolate, and contain the spread of the outbreak. Multiple studies have reported a high degree of sensitivity for chest CT in the diagnosis of COVID-19 pneumonia^7,9,13,14^. However, multiple radiology and thoracic professional associations in the US and the UK have recommended against using chest CT for screening or for the routine diagnosis of COVID-19, in part due to the potential for overlap with other high prevalence entities such as influenza pneumonia. In this study, the algorithm has a high specificity in such a setting of 93%. Sub-analysis within varying clinical indications for CT scanning demonstrated lower false positive rates in populations undergoing imaging for oncologic diagnosis and follow-up compared with acute and trauma care. Notably, this performance was consistent in the subgroup of RT-PCR confirmed influenza pneumonia, which included cases with H1N1. Thus, given the challenges in confidently distinguishing between COVID-19 associated pneumonia and other types of pneumonia, there may be a role for AI in CT-based diagnosis, characterization, or quantification of response. Performance was observed to be highest in patient cohorts from centers utilizing CT earlier in diagnostic pathway, while settings utilizing CT in cases with advanced pneumonia demonstrated poorer detection sensitivity. Further work regarding the diagnostic utility of this algorithm in the setting of early vs. advanced COVID-19 related pneumonia is warranted.

We also sought to maximize the generalizability from a technical standpoint. To reduce the effect of body habitus and extra-thoracic pathology, a lung segmentation algorithm was used to localize to chest cavity and ensure the classification algorithm was focused on the lungs and excluded the presence of factors outside the patient (such as isolation bags around the patients in Hubei). All classification models were trained utilizing an aggressive augmentation scheme to minimize over-fitting. While slice-based or hybrid 3D models demonstrated high validation accuracies, the simpler model utilizing single 3D volumes produced the most generalizable framework. Experiments testing the algorithm generalizability demonstrated 79.8% sensitivity could be achieved when the cohort of patients from Japan were excluded from training and only used in testing to evaluate model performance in an unseen domain. While the model performance exhibited a moderate decrease in sensitivity, it appeared to generalize well given the variabilities in CT acquisition and in clinical considerations across multi-nation cohorts. Prospective validation is certainly needed prior to making definitive statements on the performance of this AI system and potential improvements on slice-based and hybrid models to yield more desirable results.

There are several limitations to this study. Model training was limited to patients with positive RT-PCR testing and COVID-19 related pneumonia on chest CT in order to differentiate between COVID-19 related disease and other pathologies. However, CT is often negative despite positive RT-PCR test^21^. Given that viral infectiousness can often predate symptoms, CT plus RT-PCR is likely a more accurate and sensitive strategy than either alone, although this is somewhat speculative^11^. Delayed RT-PCR or limitations in access or availability could also make CT testing more attractive for specific subsets of patients or in a resource constrained environment, such as persons under investigation for exposure history or contact tracing, triage for resource utilization, prognosis, or to assist with isolation compliance, although this is speculative. Finally, our AI algorithm aims to classify chest CT scans as positive vs. negative for COVID-19 pneumonia and in positively classified CT scans, it delivers a saliency map for visualization of AI-associated predictions. While useful for general visualization of AI output, this does not delineate COVID-19 burden, which may be more accurately depicted by segmentation algorithms.

The test success metrics are highly dependent upon pretest background prevalence, and testing practices may vary according to exposure rates and phase of pandemic^22^. The prevalence in our constructed testing set was 24%, which may be representative of some outbreak dynamics at the time of writing. We were limited in truly evaluating the generalizability of this model to an independent population, as our positive and negative cases were derived from separate populations. It is also important to note the prevalence and testing conditions of COVID-19 positive patients varied by cohort. In Japan, the patients were a mixture of incidental Diamond Princess cruise ship exposures or community acquired COVID-19, with a diverse multinational population, but all were all PCR positive with CT lesions. In Italy, CT scans practice varied from acute care screening to mainly inpatients, or at the discretion of the treating physician, commonly later in the disease process. In Hubei Province, China, CT scans were routinely obtained on the same day as a positive RT-PCR in an acute setting/fever clinics during the initial outbreak period. RT-PCR was positive in all patients but was not a requisite for the diagnosis of COVID-19 at that time, which could be made with CT and exposure history during peak prevalence during the outbreak.

In conclusion, an AI system derived from heterogeneous multinational training data delivers acceptable performance metrics for the classification of chest CT for COVID-19 infection. While CT imaging may not necessarily be actively used in the diagnosis and screening for COVID-19, this deep learning-based AI approach may serve as a standardized and objective tool to assist the assessment of imaging findings of COVID-19 and may potentially be useful as a research tool, clinical trial response metric, or perhaps as a complementary test tool in very specific limited populations or for recurrent outbreaks settings.

## Methods

### COVID-19 study population

Patients with COVID-19 infection confirmed by RT-PCR undergoing CT evaluation for diagnosis or evaluation of infection were identified for study inclusion at four international centers: (1) 700 patients from The  Xiangyang NO.1 People’s Hospital Affiliated Hospital of Hubei University of Medicine in Hubei Province, China, (2) 147 patients from the Self-Defense Forces Central Hospital, Tokyo, Japan, (3) 130 patients from San Paolo Hospital, Milan, Italy, and (4) 16 patients from Cà Granda Ospedale Maggiore Policlinico Milano, Milan, Italy. Study inclusion criteria included positive findings for COVID-pneumonia by expert radiologist interpretation and minimum technical requirements. Summary characteristics of each institutional cohort of patients meeting study criteria are provided in Table 1. The timing of CT scan acquisition in relation to onset of COVID-19 symptoms and/or diagnosis varied and was highly dependent on regional/national standard of care. In Hubei Province, China, CT scans were routinely obtained on the same day as a positive RT-PCR in an acute setting where patients with symptoms reported for clinical assessment, as well as patients with exposure and/or travel history to high prevalence regions. In Italy, the practice and use of CT varied by hospital. Patients in the larger Milan cohort underwent CT in more of a screening-like setting at acute presentation with symptoms or exposure history at the point of care. The smaller Milan cohort of 15 patients were largely obtained in an inpatient setting. The most diverse cohort was in Japan, where patients had a mixture of incidental exposures or community acquired COVID-19. In addition, the CT acquisition parameters varied by center and within centers (Supplementary Table 3). CTs underwent a centralized evaluation by two expert radiologists for confirmation of COVID-19 associated lung disease. Local IRB and ethics and research board approvals for retrospective evaluation were obtained at each site, along with 2 way data-sharing agreements with NIH. (1) First Affiliated Hospital of Hubei University of Medicine local ethics approval #20200702150947, (2) Self-Defense Forces Central Hospital IRB #01–014, and (3) University of Milan (both cohorts from Italian hospitals) IRB #562–2020. Due to the nature of the retrospective observational study, individual informed consent was waived.

### Control study population

A balanced control population was identified from two institutions and one publicly available dataset. The control group weighed multiple clinical indications for chest CT and confounding diagnoses, such as RT-PCR or microbiology proven non-COVID-19 pneumonias from bacteria, fungi, and non-COVID viruses, as well as cancer staging and diagnosis, emergency care, and other clinical indications for chest CT imaging. These datasets are individually described, by institution and indication, in Table 1. Briefly, 972 patients undergoing non-contrast CT scans of the chest at the State University of New York (SUNY) Upstate Medical Center between 9/15/2020 and 3/15/2020, of which 949 met minimum technical considerations for inclusion. The distribution of indications for the CTs in the control group can be seen in Supplementary Table 2. In addition, 143 patients undergoing CT evaluation of laboratory-confirmed pneumonias from SUNY Upstate Medical Center were collected and characterized for use as a differential diagnosis test set, with confirmation of infection by culture (for bacterial pneumonia) or RT-PCR (for viral cases), of which 140 met minimum technical considerations for inclusion. The distribution of RT-PCR and culture data are included in Supplementary Table 4. Similarly, 36 patients at the National Institutes of Health undergoing CT evaluation of known pneumonia were collected to broaden the heterogeneity of the control group. A cohort of 102 patients with unremarkable lung findings were identified from a population of men with prostate cancer undergoing staging at the National Institutes of Health for inclusion as a non-diseased normal cohort, of which 100 met minimum technical considerations for inclusion. Local IRB and ethics approvals for retrospective evaluation and data sharing were obtained at each site: (1) NIH pneumonia cohort IRB #12-CC-0075, (2) NIH prostate cancer staging cohort IRB #18-C-0017, and (3) SUNY Upstate Medical University IRB #1578307–1. Due to the nature of the retrospective observational study, individual informed consent was waived. Finally, a total of 470 CTs were derived from the publicly available dataset LIDC (downloaded 3/25/2020). This dataset is an open-source dataset consisting of CT scans of the thorax from seven academic centers and includes lung nodules of various sizes^23^. Summary of dataset inclusion is provided in Table 1. The image characteristics of these datasets can be seen in Supplementary Table 3.

### Algorithm development

The design and workflow of the classification algorithm is shown in Fig. 1. Briefly, a lung segmentation algorithm was developed to identify and localize whole lung regions, which were then used as input for CT-based prediction of COVID-19 disease. Multiple classification models and rationales were implemented, including a hybrid model that performs 3D classification on multiple crops (i.e., several slices) at fixed resolution within an image, and a full 3D image classification implementation considering one complete volume at a fixed size.

### Lung segmentation model

The lung segmentation model was trained using the AH-Net architecture^24^. In order to address the challenges from GGO/consolidation patterns, the network trained with LIDC dataset consisting of 1018 images and 95 in-house CT volumes from a training set defined in Table 1, which had considerable amounts of GGO/consolidation observations to ensure accurate segmentation in cases with a large proportion of altered parenchyma (Supplementary Fig. 3). The in-house data were manually annotated by two expert radiologists. All images were resampled to a resolution of 0.8 mm × 0.8 mm × 5.0 mm and intensity clipped to a HU range (−1000, 500). Acceptable use was determined after the algorithm achieved mean 0.95 Dice similarity coefficient (range 0.85–0.99, std. dev 0.06) at validation.

### Image classification model

Both the hybrid 3D and full 3D models used in this study were based on a Densnet-121 architecture adapted to utilize 3D operations (i.e., 3D convolutions) compared to original 2D implementation^25^. Images were clipped to HU range (−1000, 500) and cropped to bounding box fitting to the maximum dimensions of lung regions with an extended 5 voxel buffer. For the full 3D model, the entire lung region (without masking) was resampled to size 192 × 192 × 64 for training and inference. For the hybrid model, images were resampled to resolution 1 mm × 1 mm × 5 mm and sub-crops of 192 × 192 × 32 were sampled from lung regions, applying mask to obtain lung-only tissue, at a frequency of 6 crops/patient for training and 15 crops/patient at inference. These implementations are shown in Fig. 1.

Data augmentation was performed to avoid bias of center-specific characteristics, and included image intensity and contrast adjustment, introduction of random gaussian noise, flipping, and rotation. Within each mini batch, data were sampled to ensure class balance between COVID and non-COVID groups. Attention/activation maps to visualize regions within the image utilized for prediction were generated using Grad-CAM method^26^. Algorithm development was implemented in Tensorflow and code is publicly available as part of the NVIDIA Clara Train SDK on NGC^27^.

### Statistical analysis

Classification performance was evaluated by overall accuracy, positive predictive value, negative predictive value, sensitivity, and specificity for correctly distinguishing between COVID-19 vs. any other condition. Summary statistics of false positive predictions (incorrectly labeling as COVID-19) were reported separately for pneumonia cohorts and all-comer/any-indication cohorts. Hold out test sets were identified for each model with attention to the ability to translate models across demographics and disease stages.

Reproducibility of the training schema and generalizability of resultant models were assessed by removing the cohort of COVID-19 patients from Tokyo, Japan from training and validation datasets. Models were retrained utilizing identical algorithm configuration and hyperparameters as the original models. Overall performance, as well as accuracy specific to Japan cohort, were reported and used for evaluation.

### Reporting summary

Further information on research design is available in the Nature Research Reporting Summary linked to this article.

## Supplementary information

Supplementary Information

Reporting Summary

## Data Availability

Due to the multinational nature of the datasets, restrictions on data sharing agreements are governed by each institution’s policies. At the time of publication, local IRB and ethics approvals were not obtained to allow public sharing of raw imaging data from individual centers contributing to this paper. A portion of this study utilized data from publicly available dataset LIDC (downloaded 3/25/2020; https://wiki.cancerimagingarchive.net/display/Public/LIDC-IDRI) 23. Readers are invited to contact the corresponding author for further information on data availability and data sharing policies.

## Code availability

All models presented in this work were developed using NVIDIA Clara Train platform. As such, all NVIDIA-related frameworks and models specific to this publications are available at no cost as part of the NVIDIA Clara Train SDK on NGC^27^ at https://ngc.nvidia.com/catalog/containers/nvidia:clara:ai-covid-19. This includes both inference-based pipelines for evaluation, as well as model weights for further training or fine tuning in outside institutions.
