# Supplementary material for: Artificial intelligence for the detection of COVID-19 pneumonia on chest CT using multinational datasets
Source: Nat Commun. 2020 Aug 14;11:4080. doi: 10.1038/s41467-020-17971-2 (PMC7429815; doi:10.1038/s41467-020-17971-2)
Supplement: Supplementary file 1 — Supplementary Information [file 41467_2020_17971_MOESM1_ESM.pdf]

Supplementary Table 1. Test Results by center/disease entity

		hybrid 3D			full 3D		
**Disease Cohort**	**Center**	**other**	**covid**	**Accuracy**	**other**	**covid**	**Accuracy**
COVID-19	Hubei, China	23	184	0.889	24	183	0.884
	Milan, Italy	16	38	0.704	15	39	0.720
	Tokyo, Japan	3	46	0.939	6	43	0.876
	Milan, Italy	5	10	0.667	6	9	0.6
	SUNY, USA	1	0	0.0	1	0	0.0
any clinical indication	SUNY, USA	453	47	0.906	457	43	0.914
cancer diagnosis/staging	LIDC [ref]	256	15	0.945	266	5	0.982
	NIH, USA	92	8	0.920	91	9	0.910
Pneumonia	SUNY, USA	110	30	0.786	126	14	0.900
All		*959*	*378*	*0.889*	*992*	*345*	*0.908*

Supplementary Table 2. Clinical Indications for SUNY all-comer patient population, including all patients and test-only patients with false-positive rates by 3D classification model

Indication	All Patients	Test Set	
	N(%)	N(%)	False Positive Rate by 3D classification
oncology	292 (30.7%)	165 (32.9%)	0.055
lung nodule evaluation	188 (19.8%)	74 (14.8%)	0.027
acute respiratory	145 (15.3%)	68 (13.6%)	0.103
infectious disease	59 (6.5%)	23 (4.6%)	0.087
acute cardiac	44 (4.6%)	22 (4.4%)	0.273
trauma	43 (4.5%)	22 (4.4%)	0.273
other	178 (18.7%)	127 (25.3%)	0.087
**Total**	**950**	**501**	**0.086**

Supplementary Table 3. Image Acquisition Characteristics

Disease Cohort	Center	in-plane resolution	slice thickness							KVP	Scanner Manufacturer
			t<1	1	1<t<2	2	2<t<5	5	t>5		
COVID-19	Hubei, China	0.755 (0.30-1.11)	4	17		24	1	652		80-120 KVP	TOSHIBA, GE
	Milan, Italy	0.731 (0.57-0.95)			6		120	8		120-140 KVP	GE
	Tokyo, Japan	0.729 (0.53-0.89)			7	1	3	169		70-130 KVP	TOSHIBA, GE, SIEMENS, HITACHI
	Milan, Italy	0.756 (0.56-0.98)					14	1		100-120	SIEMENS
	Syracuse, NY, USA	* see footnote						1			
any clinical indication	Syracuse, NY, USA	0.714 (0.33-1.03)	69	85	107		11	677	1	80-140 KVP	TOSHIBA, GE, SIEMENS, PHILIPS
cancer diagnosis/staging	LIDC [19]	0.703 (0.46-0.98)	74	49	124	18	203	2		110-150 KVP	TOSHIBA, GE, SIEMENS, PHILIPS
	NIH, USA	0.849 (0.67-0.98)	1	28			3	68		90-130 KVP	TOSHIBA, GE, SIEMENS
Pneumonia	SUNY, USA	0.721 (0.51-0.78)	2	4	14	1		120		80-140 KVP	TOSHIBA, GE, SIEMENS, PHILIPS
	NIH, USA	0.664 (0.47,0.93)	5	1		1	2	19	1	80/120 KVP	TOSHIBA, GE, SIEMENS, PHILIPS

Supplementary Table 4: Microbiology of Pneumonia Test Dataset

**Viral Pneumonia**		**Bacterial Pneumonia**	
Influenza A H3	22	Pseudomonas aeruginosa	8
Influenza A 2009 H1	16	MRSA	8
Influenza B	9	Staphylococcus-coagulase positive	4
Influenza A	8	Haemophilus influenzae B lactamase (-)	3
Rhinovirus/Enterovirus	5	MSSA	2
Adenovirus	2	Streptococcus pneumoniae	2
Influenza A H3 virus	2	Corynebacterium striatum	2
Parainfluenza virus 3	2	L. pneumophila serogroup 1 antigen	1
Adenovrius	1	Mycobacterium gordonae	1
CMV	1	Streptococcus Group A antigen	1
Coronavirus NL63	1	Proteus mirabilis	1
		Proteus vulgaris	1
		Mycobacterium mucogenicum	1
		Providencia rettgeri	1
		Achromobacter xylosoxidans	1
		Escherichia coli	1
		Mycobacterium intracellulare	1
		Serratia marcencens	1
		Pseudomonas flurenscens	1
		Streptococcus constellatus	1
		Streptococcus intermedius	1
		Actinomyces meyeri	1
**Total Virus**	**69**	**Total bacterial**	**44**

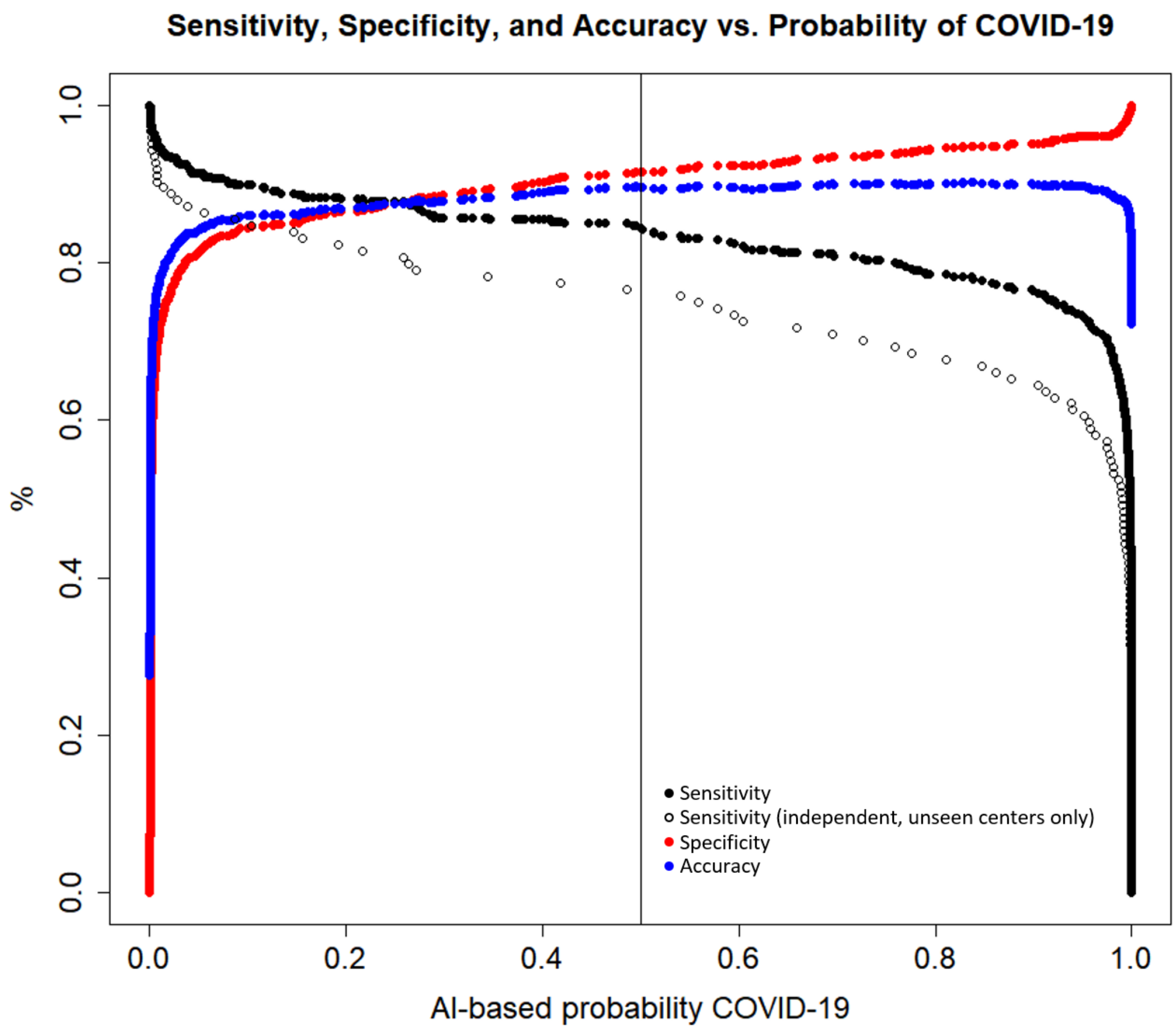

Supplementary Figure 1. Output of AI-based model for prediction of COVID-19 vs. Accuracy, Sensitivity, and Specificity from generalizability experiment with **i**ndependent testing populations from Japan and Italy for 3D model. Probability refers to output of AI model, with 0.5 (horizontal line) corresponding to threshold for determination of COVID-19 classification.

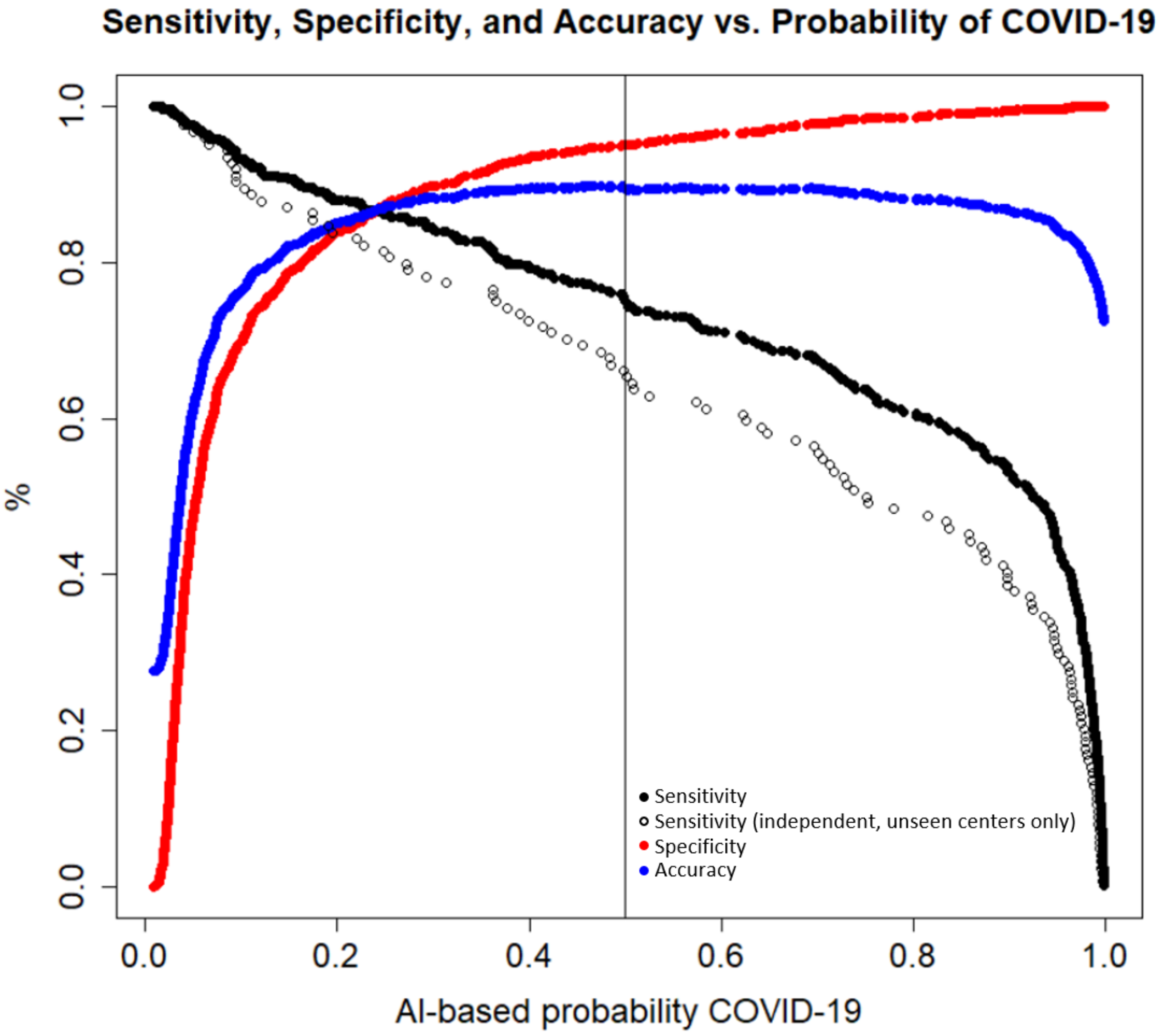

Supplementary Figure 2. Output of AI-based model for prediction of COVID-19 vs. Accuracy, Sensitivity, and Specificity from generalizability experiment with **i**ndependent testing populations from Japan and Italy for Hybrid 3D model. Probability refers to output of AI model, with 0.5 (horizontal line) corresponding to threshold for determination of COVID-19 classification.

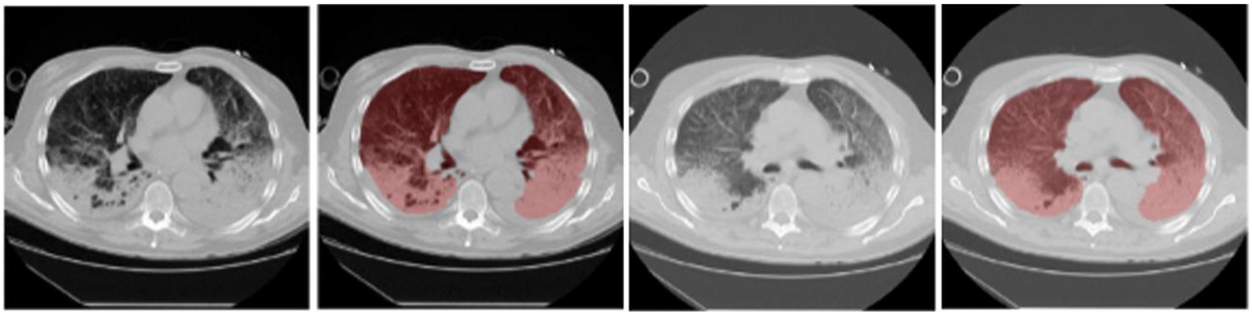

Supplementary Figure 3. Lung segmentation in two test cases with extensive consolidation related to COVID-19 pneumonia.
