# Supplementary material for: Artificial intelligence for the detection of COVID-19 pneumonia on chest CT using multinational datasets
Source: Nat Commun. 2020 Aug 14;11:4080. doi: 10.1038/s41467-020-17971-2 (PMC7429815; doi:10.1038/s41467-020-17971-2)
Supplement: Supplementary file 2 — Reporting Summary [file 41467_2020_17971_MOESM2_ESM.pdf]

nature research

Corresponding author(s): Brad Wood, Baris Turkbey

Last updated by author(s): Jul 9, 2020

Reporting Summary

Nature Research wishes to improve the reproducibility of the work that we publish. This form provides structure for consistency and transparency in reporting. For further information on Nature Research policies, see our Editorial Policies and the Editorial Policy Checklist.

Statistics

For all statistical analyses, confirm that the following items are present in the figure legend, table legend, main text, or Methods section.

n/a	Confirmed	
☐	☒	The exact sample size (n) for each experimental group/condition, given as a discrete number and unit of measurement
☐	☒	A statement on whether measurements were taken from distinct samples or whether the same sample was measured repeatedly
☐	☒	The statistical test(s) used AND whether they are one- or two-sided<br>*Only common tests should be described solely by name; describe more complex techniques in the Methods section.*
☒	☐	A description of all covariates tested
☒	☐	A description of any assumptions or corrections, such as tests of normality and adjustment for multiple comparisons
☐	☒	A full description of the statistical parameters including central tendency (e.g. means) or other basic estimates (e.g. regression coefficient) AND variation (e.g. standard deviation) or associated estimates of uncertainty (e.g. confidence intervals)
☒	☐	For null hypothesis testing, the test statistic (e.g. F, t, r) with confidence intervals, effect sizes, degrees of freedom and P value noted<br>*Give P values as exact values whenever suitable.*
☒	☐	For Bayesian analysis, information on the choice of priors and Markov chain Monte Carlo settings
☒	☐	For hierarchical and complex designs, identification of the appropriate level for tests and full reporting of outcomes
☒	☐	Estimates of effect sizes (e.g. Cohen's d, Pearson's r), indicating how they were calculated

Our web collection on statistics for biologists contains articles on many of the points above.

Software and code

Policy information about availability of computer code

Data collection	no software was used
Data analysis	All models presented in this work were developed using NVIDIA Clara Train platform. As such, all NVIDIA-related frameworks and models specific to this publications are available at no cost as part of the NVIDIA Clara Train SDK on NGC29 at https://ngc.nvidia.com/catalog/containers/nvidia:clara:ai-covid-19. This includes both inference-based pipelines for evaluation, as well as model weights for further training or fine-tuning in outside institutions.

For manuscripts utilizing custom algorithms or software that are central to the research but not yet described in published literature, software must be made available to editors and reviewers. We strongly encourage code deposition in a community repository (e.g. GitHub). See the Nature Research guidelines for submitting code & software for further information.

Data

Policy information about availability of data

All manuscripts must include a data availability statement. This statement should provide the following information, where applicable:

- Accession codes, unique identifiers, or web links for publicly available datasets
- A list of figures that have associated raw data
- A description of any restrictions on data availability

Due to the multi-national nature of the datasets, restrictions on data sharing agreements are governed by each institution's policies. At the time of publication, local IRB and ethics approvals were not obtained to allow public sharing of raw imaging data from individual centers contributing to this manuscript. A portion of this study utilized data from publicly available dataset LIDC (downloaded 3/25/2020; https://wiki.cancerimagingarchive.net/display/Public/LIDC-IDRI) 23. Readers are invited to contact the corresponding author for further information on data availability and data sharing policies.

Field-specific reporting

Please select the one below that is the best fit for your research. If you are not sure, read the appropriate sections before making your selection.

☒ Life sciences ☐ Behavioural & social sciences ☐ Ecological, evolutionary & environmental sciences

For a reference copy of the document with all sections, see nature.com/documents/nr-reporting-summary-flat.pdf

Life sciences study design

All studies must disclose on these points even when the disclosure is negative.

Sample size	2724 scans from 2619 patients were used in this study, including 1029 scans of 922 patients with RT-PCR confirmed COVID-19 and lung lesions related to COVID-19 pneumonia. No sample size calculation was made. Instead, we focused efforts to derive a representative control population of patients undergoing Chest CT for variety of clinical indications from multiple data sources. Sample size was limited by the availability of COVID-19 data. Additionally, control populations were identified from multiple datasets at multiple institutions. Here, sample size was limited to the availability of data for a given case (NIH protocols) or timeframe (SUNY protocol).
Data exclusions	Data were excluded from analysis if they did not contain the full chest cavity in the field of view. For patients with RT-PCR confirmed COVID-19, we only included scans which had positive findings associated with COVID-19 on CT evaluation.
Replication	The results of the AI algorithm in the test set are reproducible due to the nature of the algorithm, meaning if we send the exact same input into the algorithm multiple times we will get the exact same result, there is no stochastic nature to applying the algorithm to testing data. We tested the reproducibility of our results by re-training our algorithms under the exact same computational conditions but removed one center from the training population and reserved it for independent testing. This allows us to evaluate the reproducibility of our models performance and, importantly, for the generalizability of the model to new populations.
Randomization	Patients were randomly assigned to training, validation, and testing by center to avoid any center-specific bias
Blinding	Blinding is not relevant to this study, as we did not perform a radiologist vs. AI study. In other words, this study aimed to train and evaluate the performance of an algorithm using radiologist interpretation as ground truth. Future studies on the performance of AI compared to radiologists in new patient populations would require blinded evaluation.

Reporting for specific materials, systems and methods

We require information from authors about some types of materials, experimental systems and methods used in many studies. Here, indicate whether each material, system or method listed is relevant to your study. If you are not sure if a list item applies to your research, read the appropriate section before selecting a response.

Materials & experimental systems

n/a	Involved in the study	
☒	☐	Antibodies
☒	☐	Eukaryotic cell lines
☒	☐	Palaeontology and archaeology
☒	☐	Animals and other organisms
☐	☒	Human research participants
☒	☐	Clinical data
☒	☐	Dual use research of concern

Methods

n/a	Involved in the study	
☒	☐	ChIP-seq
☒	☐	Flow cytometry
☒	☐	MRI-based neuroimaging

Human research participants

Policy information about studies involving human research participants

Population characteristics	COVID-19 Study Population<br>Patients with COVID-19 infection confirmed by RT-PCR undergoing CT evaluation for diagnosis or evaluation of infection were identified for study inclusion at four international centers: 1) 700 patients from The First Affiliated Hospital of Hubei University of Medicine in Hubei Province, China, 2) 147 patients from the Self-Defense Forces Central Hospital, Tokyo, Japan, 3) 130 patients from San Paolo Hospital, Milan, Italy and 4) 16 patients from Cà Granda Ospedale Maggiore Policlinico Milano, Milan, Italy. Study inclusion criteria included positive findings for COVID-pneumonia by expert radiologist interpretation and minimum technical requirements. The timing of CT scan acquisition in relation to onset of COVID-19 symptoms and/or diagnosis varied and was highly dependent on regional/national standard of care. In Hubei Province, China, CT scans were routinely obtained on the same day as a positive RT-PCR in an acute setting where patients with symptoms reported for clinical assessment, as well as patients with exposure and/or travel history to high prevalence regions. In Italy, the practice and use of CT varied by hospital. Patients in the larger Milan cohort underwent CT in more of a screening-like setting at acute presentation with symptoms or exposure history at the point of care. The smaller Milan cohort of 15 patients

were largely obtained in an inpatient setting. The most diverse cohort was in Japan, where patients had a mixture of incidental exposures or community acquired COVID-19. CTs underwent a centralized evaluation by 2 expert radiologists for confirmation of COVID-19 associated lung disease. Local IRB and ethics approvals for retrospective evaluation and data sharing were obtained at each site.

Control Study Population
A balanced control population was identified from two institutions and one publicly available dataset. The control group weighed multiple clinical indications for chest CT and confounding diagnoses, such as RT-PCR or microbiology proven non-COVID-19 pneumonias from bacteria, fungi, and non-COVID viruses, as well as cancer staging and diagnosis, emergency care, and other clinical indications for chest CT imaging. These datasets are individually described, by institution and indication, in Table 1. Briefly, 972 patients undergoing non-contrast CT scans of the chest at the State University of New York (SUNY) Upstate Medical Center between 9/15/2020 and 3/15/2020, of which 949 met minimum technical considerations for inclusion. Additionally, 143 patients undergoing CT evaluation of laboratory-confirmed pneumonias from SUNY Upstate Medical Center were collected and characterized for use as a differential diagnosis test set, with confirmation of infection by culture (for bacterial pneumonia) or RT-PCR (for viral cases), of which 140 met minimum technical considerations for inclusion. The distribution of RT-PCR and culture data are included in Supplementary Table 4. Similarly, 36 patients at the National Institutes of Health undergoing CT evaluation of known pneumonia were collected to broaden the heterogeneity of the control group. A cohort of 102 patients with unremarkable lung findings were identified from a population of men with prostate cancer undergoing staging at the National Institutes of Health for inclusion as a non-diseased normal cohort, of which 100 met minimum technical considerations for inclusion. Local IRB and ethics approvals for retrospective evaluation and data sharing were obtained at each site. Finally, a total of 470 CTs were derived from the publicly available dataset LIDC (downloaded 3/25/2020). This dataset is an open-source dataset consisting of CT scans of the thorax from 7 academic centers and includes lung nodules of various sizes.

Hubei, China COVID cohort: 363 Male, 353 Female, Median Age 49 (18†-92)
Milan, Italy COVID cohort: 220 Male, 90 Female, Median Age 60 (18-96)
Tokyo, Japan COVID cohort: 91 Male, 60 Female Median Age 60 (4-87)
Milan, Italy COVID cohort: 10 Male, 5 Female Median Age 55 (31-85)
Syracuse, NY, USA allcomer cohort: 437 Male, 534 Female Median Age 65 (19-100)
NIH, USA prostate cancer cohort:100 Male Median Age 69 (30-89)
Syracuse, NY, USA Pneumonia cohort: 73 Male, 42 Female, Median Age 66 (13-101)
NIH, USA 28 Male, 8 Female, Median Age 21 (4-71)

Recruitment

Details regarding patient selection and characteristics are provided in the population characteristics summary above. Patient CTs and minimal clinical data analyzed in the models were transferred from 4 institution hospitals and shared with (and analyzed by) the NIH team, with data being sent by primary institutions under a standard "NIH Collaboration Agreement" between NIH and each of 4 individual institutions, under 4 separate collaboration agreements. These agreements outlined the data sharing and human subjects protections, cleared by the Office of Technology Transfer after confirmation with staff of the Office of Human Subjects Protection and the IRB. As a part of this process, the NIH Office of Technology Transfer Attestation was completed addressing the protection of human subjects for Material Transfer Agreements or Data Transfer Agreements.

Given that all COVID-19 patient cohorts were from distinct centers around the world, it is possible there is biased representation in the dataset due to the timing and use of CT in those environments. We attempted to overcome this by including several centers with diverse healthcare practices. We attempted to control for bias in the control population by also diversifying the population characteristics to patients undergoing CT for a variety of reasons that would reflect a typical hospital worflow, such as cancer-related imaging, emergency-related imaging, and pneumonia-related imaging.

Ethics oversight

Local IRB approvals were completed at each institution:
NIH - protocol 12-CC-0075 (PI: Brad Wood, M.D.) and 18-C-0017 (PI: Baris Turkbey M.D.)
Self-Defense Forces Central Hospital IRB: 01-014
Xiangyang First People's Hospital Affiliated to Hubei University of Medicine Xiangyang: local hospital ethics approval #20200702150947
University of Milan (both hospital centers) IRB: 562_2020
SUNY Upstate Medical University IRB: 1578307-1

In all IRB settings, informed consent was waived

Note that full information on the approval of the study protocol must also be provided in the manuscript.
